# Parliament and the Profession

**Published:** 1917-11-17

**Authors:** 


### Parliament and the Profession.

### Inoculation.

Mr. Lynch asked the Under-Secretary of State for War whether, in view of misconceptions as to anti-typhoid inoculation and prejudice against that form of treatment, he will consider the translation and circulation of a report on the subject communicated to the French Academy of Science by Dr. Richet, which showed the boon to the soldier as well as to the State conferred by this system of inoculation. Mr. Macpherson considered that the prejudice was negligible, and did not think the course suggested by Mr. Lynch to be necessary.

Some later questions elicited the information that 184 officers and men have died of enteric in France, and 48 of paratyphoid. 2,321 cases of typhoid (enteric) and 3,871 cases of paratyphoid have occurred. The totals for dysentery are not available. Of the deaths from paratyphoid, 39 had been inoculated, and 9 had not been inoculated. Of the deaths from enteric, 62 had been inoculated, and 122 had not been inoculated.

### Artificial Limbs.

Major Chapple sought information as to the proportion of artificial limbs returned for renewal, and was told by Sir A. Griffith-Boscawen that it is recognised that a large proportion of the men who are fitted for artificial legs have to return for the limb to be refitted within six months or a year. The refitting is necessitated by shrinking of the stump which takes place, not simply as a result of the passage of time, but as a result of the pressure of the socket of an artificial limb. The policy of supplying a provisional limb of a simple type before the fitting of the permanent limb is one on which surgical opinion is divided. It has been adopted in some cases, and its wider adoption is under consideration.

### Hospitals for Venereal Diseases.

Mr. Macpherson informed Mr. Arnold Ward that the location of military hospitals for venereal diseases is a matter of the greatest difficulty, and meets in every instance with strong local opposition. Feeling is strong against the use of buildings for the purpose, and it has become necessary to utilise hutments which are the property of the Government.

### Royal Army Medical Corps.

Mr. Macpherson informed Major Davies that medical men commissioned as officers in the Royal Army Medical Corps undergo a short course of instruction in their military duties, and much importance is attached to this training, which is not confined to drill, but includes military sanitation, anti-gas protection, and the duties of a medical officer with troops in the field. Major Davies was told by the Under-Secretary that the Committee of Inquiry upon Distribution of Medical Officers will not report on the present status of the Director-General of Army Medical Services.

### Sir William Babtie.

Mr. Macpherson explained to Major Davies that Surgeon-General Sir William Babtie was Director of Medical Services of the War Office before the Report of the Mesopotamian Commission was issued. After the appearance of the Report he was given leave pending its consideration and, after his explanation to the Army Council as to his connection with the circumstances which led to the Report had been submitted to the Army Council and found satisfactory, he was recalled from leave to resume his official duties.

